# Novel Approaches to Epigenetic Therapies: From Drug Combinations to Epigenetic Editing

**DOI:** 10.3390/genes12020208

**Published:** 2021-01-31

**Authors:** Aleksandra Majchrzak-Celińska, Anna Warych, Mikołaj Szoszkiewicz

**Affiliations:** Department of Pharmaceutical Biochemistry, Poznan University of Medical Sciences, 60-781 Poznań, Poland; anne.warych@gmail.com (A.W.); mszoszk@o2.pl (M.S.)

**Keywords:** epigenetic therapy, DNMT inhibitors, HDAC inhibitors, epigenetic editing, drug combinations, oncometabolites, multitargeting drugs, CRISPR/Cas9

## Abstract

Cancer development involves both genetic and epigenetic alterations. Aberrant epigenetic modifications are reversible, allowing excellent opportunities for therapeutic intervention. Nowadays, several epigenetic drugs are used worldwide to treat, e.g., myelodysplastic syndromes and leukemias. However, overcoming resistance and widening the therapeutic profiles are the most important challenges faced by traditional epigenetic drugs. Recently, novel approaches to epigenetic therapies have been proposed. Next-generation epigenetic drugs, with longer half-life and better bioavailability, are being developed and tested. Since epigenetic phenomena are interdependent, treatment modalities include co-administration of two different epigenetic drugs. In order to sensitize cancer cells to chemotherapy, epigenetic drugs are administered prior to chemotherapy, or both epigenetic drug and chemotherapy are used together to achieve synergistic effects and maximize treatment efficacy. The combinations of epigenetic drug with immunotherapy are being tested, because they have proved to enhance antitumor immune responses. The next approach involves targeting the metabolic causes of epigenetic changes, i.e., enzymes which, when mutated, produce oncometabolites. Finally, epigenome editing makes it possible to modify individual chromatin marks at a defined region with unprecedented specificity and efficiency. This review summarizes the above attempts in fulfilling the promise of epigenetic drugs in the effective cancer treatment.

## 1. Introduction

The field of epigenetics has rapidly evolved over the last decade, bringing us novel insights into the mechanisms by which gene expression is regulated in health and disease states. A growing body of evidence has shown the importance of aberrant epigenetic alterations for cancer initiation and progression. Cancer cells are characterized by global epigenetic reorganization resulting in the CpG-specific hypermethylation of tumor suppressor gene promoters and global DNA hypomethylation at microsatellite regions, repetitive sequences, and oncogene promoters [1]. Point mutations, translocations, amplifications, and deletions in genes encoding epigenetic enzymes are also frequent in cancer [2]. Altered cancer metabolism and the production of oncometabolites influences the epigenome and contributes to the malignant phenotype of cancer cells. All of these alterations are used by cancer cells, to develop resistance to immune surveillance and chemotherapy [3].

The complex interplay between epigenetic phenomena depends on three families of epigenetic proteins, namely writers, readers, and erasers [4,5]. Writers introduce various chemical modifications on DNA and histones, readers identify and interpret those modifications, whereas erasers remove the marks. In fact, all of these epigenetic regulators can be targeted by epigenetic drugs. Today, several epigenetic drugs have been introduced into the clinic to treat cancer, and many more are being investigated in clinical trials. 5-azacytidine and 5-aza-2′-deoxycytidine, which are currently used as first-line treatment for patients with, e.g., myelodysplastic syndrome, inhibit DNA methyltransferases DNMT1 and DNMT3B, regarded as writers. Histone deacetylases (HDACs), functioning as erasers, are targeted by, e.g., vorinostat (SAHA) and romidepsin. Bromodomain (BET) inhibitors, representing drugs that target epigenetic readers, are still in clinical trials.

Despite the unprecedented investment in the development, characterization, and translation of basic knowledge into epigenetic therapies, several problems still need to be solved in order to maximize their efficacy. The most crucial are primary and secondary resistance as well as rare responses in solid tumors. The lack of locus specificity and global as well as non-chromatin effects are also a considerable drawback of conventional epigenetic drugs. In order to solve these problems, it is necessary to identify more efficient approaches in epigenetic drug discovery, as well as to develop better combination therapy of already existing drugs.

This review summarizes the new insights into epigenetic therapies in clinical oncology, taking into account the crosstalk between tumor epigenetics, immunity, and metabolism. The advent of CRISPR/Cas9 technology, which has revolutionized the field of epigenetic editing, is also highlighted. 

## 2. DNMT Inhibitors

DNA methylation is a covalent modification introduced to the cytosine at the fifth position of its carbon ring (5-mC), mostly in a CpG dinucleotide context [6]. Methylation occurs mainly at CpG islands, which are defined as stretches of DNA of 500–1500 bp long, with at least 50% of cytosines and guanines, and an observed CpG/expected CpG in excess of 0.6 [7,8]. Approximately 29,000 CpG islands can be found in the human genome, most often located in the promoter regions, close to the transcription start site or first exons. Other areas found to harbor CpG methylation include repetitive sequences, such as centromeres and transposon elements, CpG island shores, noncoding regions (i.e., enhancer regions and miRNAs), and gene bodies (leading to the silencing of alternative transcription start sites) [9]. Moreover, methylation can also be found in the X chromosome (leading to its inactivation) and is responsible for genomic imprinting.

The process of DNA methylation is regulated by the DNMT family of enzymes by the transfer of a methyl group from S-adenosyl-L-methionine (SAM) to cytosine [10]. DNMT are classified into three distinct, highly conserved families: DNMT1, DNMT2, and DNMT3 (consisting of DNMT3a, DNMT3b, and DNMT3L). DNMT1 is the most abundant DNMT in adult cells. While DNMT1 primarily functions in the maintenance methylation during replication, both DNMT3A and DNMT3B are essentially responsible for de novo methylation of either unmethylated DNA or hemimethylated DNA [11]. DNMT2 and DNMT3L are not regarded as catalytically active DNA methyltransferases.

The dynamic nature of DNA methylation enables cellular programming, homeostasis, and environmental adaptation. DNA methylation, together with histone post-translational modifications is regarded as a major regulator of chromatin structure and function. It is involved in the maintenance of genomic integrity, gene transcription, and alternative splicing. Depending on various endogenous and exogenous stimuli, DNA methylation guides gene expression according to the constantly changing environment. Thus, the primary biological functions of DNA methylation in the CpG islands is the selective exposure of gene promoters to transcription factors. Methylation at a promoter region prevents the binding of RNA polymerases and/or transcription factors, thereby inhibiting DNA transcription. However, the molecular functions of DNMTs are not limited to gene silencing and can also include transcriptional activation and post-transcriptional regulation [12]. The latter function is mediated by DNMT2-dependent RNA methylation [13].

DNA methylation is essential for fundamental processes like embryonic development or differentiation. Using CRISPR/Cas9 genome editing, Liao and coworkers have shown that targeted deletion of DNMT1 in human embryonic stem cells causes global demethylation and is lethal [11]. Tissue-specific profiles of gene expression are also maintained by DNA methylation.

However, aberrant expression and/or activities of DNMTs are involved in several pathologies, including cancer [14,15,16]. In cancer cells, tumor suppressor genes are transcriptionally silenced by promoter DNA hypermethylation, which is accompanied by global hypomethylation [17,18,19]. This aberrant methylation pattern can be caused by multiple reasons. Overexpression or enhanced catalytic activity of DNMT1, DNMT3a, and DNMT3b has been observed in multiple cancers, including acute myeloid leukemia (AML), chronic myeloid leukemia (CML), glioma, and breast, gastric, colorectal, hepatocellular, pancreatic, prostate, and lung cancers [20]. Moreover, DNA methylation makes cytosine more susceptible to spontaneous deamination, leading to C > T transition [21]. Methylated CpG changes into a TpG and, if not corrected by the repair system, results in a mutation. Indeed, many hotspot tumor mutations are found at methylated CpG sites [21]. 

Currently, two DNMT inhibitors (DNMTi), the nucleoside analogues, 5-azacitydine (azacitidine) and 5-aza-2′-deoxycytidine (decitabine), have been approved by Food and Drug Administration (FDA) and the European Medicines Agency (EMA) against myelodysplastic syndromes (MDS), AML, and chronic myelomonocytic leukemia (CMML). Following cellular uptake, nucleoside analogues are incorporated into DNA. Because of this, the cell must be in S phase of the cell cycle at a time of DNMTi exposure [21,22]. At that point, DNMTs recognize azacytosine–guanine dinucleotides and catalyze the methylation reaction by forming a covalent bond with the cytosine ring [23,24]. However, azacytosine has nitrogen instead of carbon at position 5; thus, the covalent bond cannot be broken, which results in DNMT inactivation. Besides depletion of DNMTs and a decrease in DNA methylation levels, nucleoside analogs induce DNA double-strand brakes and apoptosis [12]. They also stimulate immune response through the viral defense pathway by means of inducing the reactivation of endogenous retroviral elements [21].

The major drawback of DNMTi is that they are unsuitable for the precise targeting of a particular methylated CpG [16]. Nevertheless, their global mode of action can have positive effects. DNMTi, in addition to their abilities to reactivate genes, such as tumor suppressors, induce the expression of thousands of transposable elements, including endogenous retroviruses (ERVs) and latent cancer testis antigens (CTAs), normally silenced by DNA methylation in most somatic cells [21]. Activation of CTAs and ERVs can potentially give rise to the presence of neoantigens in treated cancer cells, thus increasing visibility to the immune system [25]. Moreover, activation of other transposable elements such as Alu elements and long interspersed elements (LINEs) can lead to a state of viral mimicry in which the treated cancer cells interpret the induced expression as being caused by an exogenous viral infection, mounting an innate immune response. Thus, the viral mimicry leads to the production of type I and type III interferon and other cytokines, as well as the attraction of cytotoxic T lymphocytes to the tumor microenvironment [26,27].

Unfortunately, azacitidine and decitabine are relatively non-specific with low chemical stability, confer significant toxicities, and require incorporation into DNA to exert their effects as covalent inhibitors [28]. Thus, even though DNMTis are now the mainstay for therapies for AML and MDS as single agents, not all patients benefit from their use as monotherapies [21]. They are also not proven to be effective in the treatment of solid tumors as monotherapy [21].

Next-generation DNMTis include guadecitabine, which is a dinucleotide prodrug for decitabine. It combines decitabine with guanosine in a single molecule. Longer half-life and better bioavailability make guadecitabine well tolerated in patients with MDS [29]. Guadecitabine has already been tested in nearly 40 clinical trials, including phase III trials. Table 1 presents ongoing clinical trials including DNMTi and other anticancer agents.

Novel nucleoside and non-nucleoside DNMTis are constantly being developed and tested [30]. The latter are commonly found in natural sources. In this regard, several polyphenols, flavonoids, antraquinones, and others were found to be able to inhibit DNMTs activity and/or expression [31,32]. Their evaluation in the context of cancer chemotherapy and chemoprevention is ongoing, and the results are promising [31,32].

## 3. Drugs Targeting Histone Modifications

Histone post-translational modifications are another major type of epigenetic mechanisms that occurs on specific amino acid residues of the histone proteins. The types of modifications that can occur in histone tails include acetylation, methylation, phosphorylation, ADP-ribosylation, deimination, isomerization, ubiquitination, parylation, citrullination, and sumoylation [33]. These modifications have been linked to dynamic changes in chromatin structures, having an impact on, e.g., transcription, replication, and DNA repair. They are also potential anticancer drug targets. 

Among the abovementioned post-translational histone modifications, acetylation, and methylation of lysine residues on H3 and H4 have been most intensively studied. The so called “charge neutralization model” explains the mode of action of histone acetylation. According to this model, a positive charge of lysine residues on H3/H4 facilitates a tight packaging of negatively charged DNA with histones. The addition of an acetyl group can loosen up the tight chromatin compaction, enabling the access of transcription factors and allowing DNA transcription [34].

Unlike histone acetylation, the effect of histone methylation on gene expression is far more complicated and depends on the targeted sites. Three lysine methylation states can be distinguished - mono-, di-, and trimethylation (me1, me2, and me3, respectively), none of which changes the electronic charge of the amino-acid side chain [35]. Thus, it is not the methylation groups themselves that influence the gene expression, but the actions are rather exerted by the chromatin effector molecules (“readers”) recognizing the methylated residues and causing the recruitment of other molecules to alter the chromatin and/or transcription states [36]. Generally, H3K4, H3K36, and H3K79 methylations are associated with gene activation, whereas H3K9, H3K27, and H4K20 methylations are thought to be associated with gene repression due to silenced chromatin states [35]. Since histone methylation-related proteins (methyltransferases, demethylases, and methyl-lysine-binding proteins) are deregulated in cancer, they are studied as potential drug targets [36]. The most relevant, in terms of anticancer therapy are as follows: H3K79 methyltransferase DOT1L, H3K4 targeting mixed lineage leukemia (MLL) and lysine-specific demethylase 1 (LSD1), and H3K27 methyltransferase EZH2 [37].

### 3.1. Histone Acetylation Modifiers

Reversible histone acetylation and deacetylation play a crucial role in gene regulation. The processes of acetylation and deacetylation are catalyzed by histone acetyltransferases (HATs) and histone deacetylases (HDACs), respectively [33]. HATs transfer an acetyl group from acetyl CoA to form ε-N-acetyl-lysine, whereas HDACs remove acetyl groups from histone tails [38]. Importantly, histone acetylation is associated with relaxed chromatin structure, making target genes more accessible for transcription factors and leading to their unconstrained expression. On the contrary, histone deacetylation ensures chromatin condensation and transcriptional repression. Thus, HATs and HDACs are associated with hyperactivity and hypoactivity of genes, respectively. Moreover, a variety of non-histone proteins, e.g., transcription factors, DNA repair enzymes, and nuclear and cytoplasmic proteins, undergo acetylation/deacetylation processes catalyzed by HATs and HDACs, respectively. In cancer, the balance of acetylation and deacetylation of lysine residues of histones and non-histone proteins is disturbed, making these epigenetic enzymes natural targets for epigenetic therapy.

The mechanisms by which HDACs contribute to cancer are diverse. It has been shown in numerous studies that overexpression of HDACs results in tumor cell proliferation, angiogenesis, metastasis, resistance to apoptosis, and alteration of the cell cycle [38]. These actions are a result of oncogenic pathways activation due to the diminished expression of tumor suppressor genes and/or activation of oncogenes [39]. Furthermore, in cancer, HDACs are characterized by aberrant recruitment to co-repressor complexes, such as NuRD (nucleosome remodeling and deacetylation), CoREST (co-repressor for element-1- silencing transcription factor), or SMRT (silencing mediator of retinoid and thyroid receptors) [40].

In recent years, HDACi have become important biologically active compounds for the treatment of cancers, in particular, hematological. Based on their homology to the yeast analogs, HDACs are divided into four classes [41]. Class I includes HDACs 1, 2, 3, and 8. Class II can be further divided into two classes: IIa (including HDAC 4, 5, 7, and 9) and IIb (HDAC 6 and 10). Class III includes sirtuins (SIRT1–SIRT7), and finally, class IV contains a single HDAC (HDAC11) with a catalytic domain shared with classes I/II HDACs. Furthermore, HDACi, based on their mechanism of action, can be divided into two groups—one acting on all HDAC classes (however not including sirtuins), called pan-HDAC inhibitors (pan-HDACi), and the other, acting on and targeting a specific class of HDACs (selective HDACi) [41]. HDACi can also be grouped into five classes based on their chemical structure: hydroxamates, cyclic peptides, short chain fatty (aliphatic) acids, benzamides, and sirtuin inhibitors [38]. The major mechanisms of action of HDACi involve the cell cycle arrest by p53-dependent or -independent induction of the cyclin-dependent kinase inhibitor p21CIP/WAF1; downregulation of oncogenes, e.g., c-MYC and c-SRC; enhanced ROS formation; and autophagy induction [42]. Moreover, HDACis inhibit metastasis by reducing the expression of genes involved in angiogenesis, migration, epithelial-to-mesenchymal transition, and cell survival, while increasing the expression of genes involved in apoptosis [43]. Finally, HDACis alter the expression of molecules that upregulate the immune system (such as MHC and costimulatory molecules), which in turn upregulates antigen presentation, resulting in T-cell activation [44].

Several HDACi have been approved by the FDA. In 2006, vorinostat (SAHA) was approved for the treatment of cutaneous T-cell lymphoma (CTCL) [45]. Belinostat was approved in 2014 for the treatment of peripheral T-cell lymphoma (PTCL). Panobinostat (LBH-589), which is a pan-inhibitor of HDACs types I, II, and IV [44], was approved for the treatment of multiple myeloma in 2015. All three abovementioned drugs contain a hydroxamic acid moiety that can bind to the zinc atom, a component in the catalytic sites of HDACs, thus inactivating HDACs [34]. The use of romidepsin (FK2280) in CTCL and PTCL therapy was approved in 2009 and 2011, respectively. Romidepsin is a member of cyclic peptide HDACi. It is a prodrug with the disulfide bond undergoing reduction by glutathione to release a zinc-binding thiol within cells. Then this thiol interacts with zinc ions in the active site of class I and II HDAC enzymes, resulting in inhibition of its enzymatic activity [34]. Moreover, chidamide (tucidinostat) was approved in China in 2014 for the treatment of PTCL. Other HDACi, tested currently in phase I or II clinical trials include pracinostat, givinostat, resminostat, abexinostat, entinostat, quisinostat, etc. [34]. The clinical trials testing combinations of HDACi and other anticancer agents are presented in Table 2, whereas the timeline of FDA approval of both HDACi and DNMTi is presented in Table 3.

Unfortunately, drug resistance to HDACi has also been observed in hematological malignancies. Several mechanisms, including drug efflux, chromatin alterations, upregulation of oxidative stress response mechanism, defects, or upregulation in apoptotic pathways, have been implicated in HDACi resistance [46]. These obstacles could be resolved, at least in part, by combining HDACi with other anticancer drugs [38]. Synergistic effects were observed when combining HDACi with, e.g., topoisomerase inhibitors, PARP inhibitors, proteasome inhibitors, radiotherapy, antimetabolites, mTOR inhibitors, or monoclonal antibodies [38]. The novel approach in HDACi design and testing also involves the design of dual inhibitors, with two active motives targeting different epigenetic proteins within the same molecule [47]. Such hybrid inhibitors have been shown to improve the therapeutic management of cancer [48]. For instance, dual EZH2/HDAC inhibitor, designed recently by Romanelli et al. [49], impaired cell viability of several cancer cell lines. It also provided G1 arrest, induced apoptosis, and increased differentiation in leukemia U937 and rhabdomyosarcoma RH4 cells. Moreover, it hampered epithelial to mesenchymal transition in glioblastoma U87 cells [49].

### 3.2. Targeting Histone Methyltransferases

Lysine histone methyltransferases (KMTs) add post-translationally one to three methyl groups to lysine residues in proteins [47]. As mentioned before, lysine methylation can either activate or silence gene transcription, depending on the specific lysine residue involved.

Unlike DNMTi and HDACi, which are also called broad “reprogrammers”, the inhibition of enhancer of zeste homologue 2 (EZH2), a histone methyltransferase that targets Lys27 of histone H3, is regarded as targeted therapy [21]. EZH2 is a member of the Polycomb group of transcriptional repressors, and its inhibitors are currently being tested in clinical trials [50]. EZH2 inhibitors can be used to treat EZH2 gain of function mutations in lymphomas. 

In January 2020, FDA approved an EZH2 inhibitor—tazemetostat—for the treatment of adults and pediatric patients aged 16 years and older with metastatic epithelioid sarcoma that are not eligible for complete resection.

An inhibitor of histone methyltransferase DOT1L activity, pinometostat, is another promising agent that is not yet approved by the FDA but is extensively studied in clinical trials [51]. Patients with acute myeloid leukemia frequently harbor rearrangements of MLL protein (MLL-r) in complexes that contain the histone methyltransferase DOT1L, leading to abnormal methylation of lysine 79 of histone H3 at MLL target genes [52]. Treatment with pinometostat results in inhibition of H3K79 methylation and hence MLL-fusion genes expression [52]. So far, the clinical trials have demonstrated the therapeutic potential for targeting DOT1L in MLL-r leukemia patients by pinometostat.

## 4. Combining DNMTi and HDACi

Since epigenetic phenomena, such as DNA methylation and histone post-translational modifications, often work in parallel as self-reinforcing systems, much attention is now being paid to testing combinations of DNMTi with HDACi, as this approach may increase the efficacy of each of the single agents. Multiple clinical trials are now testing this combination. It has been shown that HDACi given after low doses of 5-azacitidine or decitabine can augment the latter’s effect of re-expressing hypermethylated genes; however, HDACis alone are not effective in this regard [4]. As presented by Jones et al. [21], the combination of DNMTi and HDACi can increase the expression levels of tumor suppressor genes, endogenous retroviruses, and miRNAs, which might be relevant to patient response. Importantly, the combination of DNMTi and HDACi was also demonstrated to be beneficial in patients harboring solid tumors, e.g., advanced breast cancer [53] and metastatic lung cancer [54]. Moreover, the most recent report of Lu et al. [55] provides evidence that a combination of low-dose DNMTi and HDACi may permit an adjuvant approach to cancer therapy, inhibiting metastases of solid tumors. In this regard, they showed that low-dose adjuvant 5-azacytidine and entinostat, disrupt the premetastatic microenvironment and inhibit both the formation and growth of lung metastases. This effect is mediated through the selective effect on myeloid-derived suppressor cells, which are considered to be the key factors in the formation of the premetastatic microenvironment after primary tumor resection.

Another rationale for the combination of DNMTi and HDACi is that such a co-treatment also stimulates the immune system to combat cancer cells. Further, it was found that the combination of DNMTi (azacitidine) and HDAC6i (NextA) resulted in an amplified type I interferon response in human and mouse ovarian cancer cell lines [56]. The observed changes in immune response also involved the increased cytokine and chemokine expression and higher expression of the MHC I antigen presentation complex.

## 5. Epigenetic Drugs and Chemotherapy

Combination of conventional therapy or innovative anticancer treatments with epigenetic drug may offer an alternative to classical chemotherapy and may improve therapeutic effect. Epigenetic drugs, such as DNMTi and HDACi, can increase the chromatin accessibility to chemotherapeutic drugs through chromatin decompaction [57]. Preclinical and clinical studies show substantial benefits of combining several DNMTi and HDACi with diverse chemotherapeutic drugs, particularly in hematological, but also in solid malignancies. The advantages of such combinations, as compared to standard chemotherapy, are the following: Epigenetic drugs can be used for priming cancer cells for chemotherapy by chemosensitization and immunopotentiation of cancer cells; epigenetic drugs can have synergistic effects with other anticancer therapies, or they can be used to reverse acquired therapy resistance.

One of the mechanisms by which pretreatment (priming) with an epigenetic drug can increase the efficacy of chemotherapy is the reactivation of tumor-suppressor genes [58]. A phase I study of epigenetic priming with azacitidine prior to standard neoadjuvant chemotherapy with epirubicin, oxaliplatin, and capecitabine administered to patients with resectable gastric and esophageal adenocarcinoma revealed that such an approach is well tolerated and may augment chemotherapy efficacy as some of the six loci analyzed were successfully demethylated by the priming [59]. Additional mechanisms by which epigenetic priming improves the sensitivity of gastric cancer cells to chemotherapy were revealed by Moro et al. [59]. In this study, the authors demonstrated that epigenetic priming with decitabine could improve the sensitivity of gastric cancer cells to SN38 (an active metabolite of irinotecan) and cisplatin by the reactivation of apoptosis-related genes, such as *RUNX3*, *PYCARD*, *TNF*, *FAS*, and *FASLG*. Moreover, the epigenetic drugs can be used as priming agents to facilitate the transition of the tumor microenvironment from “cold” to “hot”, and potentially augment immune response, as it is being tested now with regard to the immune check-point blockade therapies [21]. Some examples of clinical trials involving epigenetic priming can be found in Table 1.

Combining epigenetic drugs with chemotherapy can have synergistic effects and can re-sensitize resistant tumor cells to radiotherapy and chemotherapy. Ovarian cancer can be used as an example showing how such combinations of epigenetic drugs and chemotherapy work. Ovarian cancer treatment with cisplatin can induce hypermethylation of multiple genes (e.g., *MEST*, *MLH1*, and *MDK*), leading to the acquired resistance phenotype [34,60]. It has been demonstrated that the addition of decitabine can abate and even reverse the resistance to cisplatin via the reactivation of those epigenetically-silenced genes [34,60]. A synergistic effect between decitabine and platinum analogs (carboplatin and cisplatin) has also been observed by Qin et al. [61]. Using YB5 cells (a clonal derivative of the SW48 colon cancer cell line), the authors presented a possible mechanism exerted by the combination of these two drugs, which is the reduction in heterochromatin protein 1α (HP1α) levels in the nucleus and chromatin remodeling. 

Besides DNMTi, also HDACi have demonstrated synergistic or cumulative anticancer effects when combined with various antitumor agents, including 5-fluorouracil, gemcitabine, docetaxel, and cisplatin [44]. In this regard, a randomized phase III placebo-controlled study revealed that hydralazine and valproate added to cisplatin and topotecan demonstrate a significant advantage in progression-free survival in advanced cervical cancer patients [62]. A more recent study confirms that, in this type of cancer, the synergistic anticancer effect also exists when panobinostat and topoisomerase inhibitors, topotecan, and etoposide are used [63]. The study shows that this effect is mediated through reactive oxygen species generation and intrinsic apoptotic pathway induction.

Regarding panobinostat, it was also demonstrated that it sensitized, to varying degrees, non-small-cell lung cancer cell lines A549, NCI-H460, and HCC827 to the antiproliferative and differentiating effects of all-trans retinoic acid [64]. Furthermore, panobinostat showed synergistic effects with zoledronic acid in a model of prostate cancer and also multiple myeloma. The observed effects were mediated by increasing reactive oxygen species generation and modulating mevalonate and p38-MAPK pathways [65]. For the treatment of multiple myeloma, the combination of panobinostat and irreversible proteasome inhibitor carfilzomib was also tested [66]. Such a combination of drugs led to synergistic inhibition of cell proliferation, resulting from an increased mitochondrial injury, caspase activation, and apoptosis induction. A clinical trial of panobinostat with carfilzomib and dexamethasone for relapsed/refractory multiple myeloma is ongoing (ClinicalTrials.gov Identifier: NCT03256045).

Using in vitro and in vivo models of non-small-cell lung cancer and patient-derived lung-cancer stem-like cells Del Bufalo [67] showed a highly synergistic interaction of a pan-HDACi givinostat (ITF2357) and pemetrexed (multi-target folate antagonist). Interestingly, the sequence of drug administration was important. The best effects were obtained when pemetrexed was followed by givinostat. This combination induced a synergistic effect, reducing cell viability and inducing apoptosis and autophagy in all cell-line models tested. On the other hand, the inverse sequence had additive to slightly synergistic growth-inhibitory effects but only in certain cell lines, whereas simultaneous administration of both drugs achieved antagonistic effects. In another study, givinostat enhanced in vitro doxorubicin cytotoxicity in both established and patient-derived sarcoma cells [68]. Givinostat reduced human sarcoma cell growth and induced apoptosis by activating the mitochondrial apoptotic pathway. Furthermore, the results were also confirmed in vivo: Combination treatment strongly impaired xenografts’ tumor growth, as compared to single treatments. The examples of clinical trials investigating the combinations of HDACi and chemotherapy are presented in Table 2.

## 6. Epigenetic Drugs in Combination with Immunotherapy

Accumulating evidence shows that combining epigenetic drugs targeting histone deacetylation or methylation with immunotherapeutics is beneficial. It has been shown that DNMTi and HDACi modulate the immune response and overcome acquired resistance to immunotherapy [69]. The mechanisms by which such combined therapies exert their actions are still awaiting elucidation, but it is suggested that the reactivation of tumor-surface antigens, endogenous retroviruses, and proteins for the major complex of histocompatibility could be mediators of the increased tumor visibility to the host immune system [3,70]. As far as DNMTis are concerned, it has been shown that they themselves can act to increase the immunogenicity of cancer cells, reshape the immune tumor microenvironment, and directly reprogram immune cells [21]. When combined with immunotherapies, they can act synergistically on both the cancer cells and immune cells to enhance antitumor immune responses [21]. Post-translational modification of histones may also regulate the behavior of cells involved in the immune response, including dendritic cells, regulatory T cells, effector T cells, myeloid-derived suppressor cells, and others [69]. Thus, HDACi can be used as priming modulators of immunotherapy. 

Recently, the combination treatment of HDACi with immune checkpoint inhibitors is being widely investigated and has promising results in several cancer types. In this regard, Knox et al. [71] showed a significant improvement of antitumor immune responses when combing anti-PD-1 and ultra-selective HDAC6i Nexturastat A. According to this study, tumor growth, along with tumor-infiltrated cells, and cytokine milieu were modified as a result of this combination treatment, making it more susceptible to immunotherapy. Eventually, this treatment modality significantly reduced tumor growth in syngeneic melanoma tumor models. HDAC inhibition was also shown to potentiate immunotherapy in another melanoma model [72], but also in triple-negative breast cancer [73], multiple myeloma [74,75], and B-cell lymphomas [76], among others. 

Moreover, experiments on mouse models have proven that targeting other epigenetic modifiers, such as SET domain, bifurcated 1 (SETDB1), lysine demethylase 1 (LSD1; also known as KDM1A), and cyclin-dependent kinase 9 (CDK9), induces viral mimicry responses and synergizes with PD1 blockade [77,78,79]. Besides the abovementioned preclinical studies, the clinical trials elucidating safety and efficacy of combined epigenetic drugs with anti-PD1/PDL1 therapy and other immunotherapy treatments are currently ongoing (Table 1 and Table 2).

## 7. Multitargeting Epigenetic Drugs

Another new approach to epigenetic therapies is the design of multitargeting epigenetic agents—molecules, which deliberately target two, or more unrelated cellular targets with high affinity (with at least one being the epigenetic enzyme) [80]. The rationale for combining multiple actions in one drug is that such an approach can simplify the treatment regimens, decrease adverse drug reactions, and reduce the potential mechanism(s) of drug resistance [81]. At the moment, inhibition of zinc-dependent HDACs is the most commonly used way of addressing more than one cancer-related target [80]. Moreover, methyltransferase and demethylase enzymes, as well as the acetyllysine-binding bromodomains, are also commonly targeted [82,83].

The long history of clinical trials exploring the simultaneous inhibition of HDACs and protein kinases led to the idea of combining the two actions into one drug. In this regard, Zang et al. [84] showed that HDACi combined with pazopanib (an inhibitor of the vascular endothelial growth factor receptor (VEGFR), platelet derived growth factor receptor, c-KIT, and fibroblast growth factor receptor tyrosine kinases) exhibits desired antitumor effects. In another study, dual inhibitors of HDAC and epidermal growth factor receptor (EGFR) were designed and synthesized based on the structure of osimertinib, the approved (EGFR) inhibitor [85]. The study showed that some of the designed compounds were even more potent in total HDAC inhibition, as compared to the approved HDAC inhibitor SAHA (Vorinostat). Nevertheless, their potency against EGFR was regarded as moderate to low [85]. Dual Janus kinase (JAK)-HDAC inhibitors, based on ruxolitinib with vorinostat, were also designed [86]. As reported by Yao et al. [86] the preferred pyrazole substituted pyrrolopyrimidine (compound number 24), inhibited JAK1 and HDACs 1, 2, 3, 6, and 10 with IC_50_ values of less than 20 nM, was <100 nM potent against JAK2 and HDAC11, and was selective for the JAK family against a panel of 97 kinases. This compound was shown to possess antiproliferative potency in hematological cell line models. Other targets that have been combined with HDAC inhibition also include cyclin-dependent kinases, casein kinase, mTOR kinase, etc. [80].

Although the vast majority of multitarget epigenetic drugs involves HDACi, the attempts to inhibit other epigenetic enzymes have also been considered. In this regard, Rabal and coworkers [83] designed and synthesized chemical probes that inhibit the activity of two epigenetic targets, histone 3 lysine 9 methyltransferase (G9a) and DNMT. They showed the antitumor efficacy of such combinations using human AML xenograft mouse model. Another popular strategy for dual-acting epigenetic agents is the combination of kinase inhibition with bromodomain binding [80]. Wang et al. [87] synthesized a potent dual Polo-like kinase 1 (PLK1) and bromodomain 4 (BRD4) inhibitor. Compound 9b exhibited good potency for both PLK1 (IC_50_ = 22 nM) and BRD4 (IC_50_ = 109 nM), as well as favorable antiproliferative activity against a panel of cancer cell lines. Its efficacy was also proved in a MV4-11 mouse xenograft model, where it exhibited favorable in vivo antitumor activity with 66% tumor growth inhibition at a dose of 60 mg/kg, with no obvious toxicity [87].

Even though the idea of multitargeting epigenetic compounds is relatively new, many such molecules are now being synthesized and tested. Of these, five candidates have successfully reached the clinical trials. The first epigenetic multitarget inhibitor which entered the clinical trial phase was CUDC-101, inhibiting EGFR, human epidermal growth factor receptor 2 (HER2), and HDAC. It is now being tested in patients with advanced head and neck, liver, breast, gastric, and non-small-cell lung cancer tumors (ClinicalTrials.gov Identifier: NCT01171924). Another interesting agent is fimepinostat (HDAC/phosphoinositide 3-kinase inhibitor). It is being tested in children and young adults with newly diagnosed diffuse intrinsic pontine glioma (DIPG), recurrent medulloblastoma, or recurrent high-grade glioma (ClinicalTrials.gov Identifier: NCT03893487). Tinostamustine is the first-in-class alkylating HDACi fusion molecule, which is now being evaluated in terms of safety and the antitumor activity in patients with newly diagnosed glioblastoma with unmethylated *MGMT* promoter (ClinicalTrials.gov Identifier: NCT03452930). The next drug candidate currently being tested in three clinical trials is HDAC/tubulin inhibitor—domatinostat (ClinicalTrials.gov Identifier: NCT04393753, NCT04133948 and NCT03812796). Domatinostat is being evaluated in terms of its safety and efficacy in patients with gastrointestinal cancers, melanoma, and advanced merkel cell carcinoma. The final compound being tested is vafidemstat (ORY-2001), which is a dual lysine-specific histone demethylase (LSD1)/monoamine oxidase B (MAO B) inhibitor. It is being tested in mild-to-moderate Alzheimer’s disease (ClinicalTrials.gov Identifier: NCT03867253).

## 8. Targeting the Metabolic Causes of Epigenetic Changes—The Role of Oncometabolites

A new area for anticancer drug development has been opened after the discovery that mutations in metabolic enzymes are related to aberrant epigenetic changes in cancer cells. Genetic mutations of the enzymes involved in the tricarboxylic acid (TCA) cycle lead to the production of small molecule metabolites involved in cancer formation, also known as oncometabolites. TCA cycle genes, encoding isocitrate dehydrogenase (*IDH1* and *IDH2*), fumarate hydratase (*FH*), and succinate dehydrogenase (*SDHA*, *SDHB*, *SDHC*, *SDHD*, and *SDHAF2*) are mutated both germinally and somatically in a number of human cancers. Mutations in *SDH* lead to the accumulation of succinate, mutations in *FH* lead to the accumulation of fumarate, whereas mutations in *IDH* result in an accumulation of (R)-2-hydroxyglutarate (2HG) [88]. All of the abovementioned products act as oncometabolites, since their chronic accumulation alters the epigenetic landscape of the cell, activating oncogenic signaling cascades. It is now evident that oncometabolites influence global patterns of both DNA methylation and histone modifications, having a huge impact on gene expression patterns. The mechanism of epigenetic changes triggered by oncometabolites involves the competitive inhibition of α-ketoglutarate (αKG)-dependent dioxygenases. In mammalian cells, this superfamily consists of >60 enzymes involved in fatty acid metabolism, oxygen sensing, collagen biosynthesis, and modulation of the epigenome [89]. All αKG-dependent dioxygenases require oxygen and αKG as co-substrates and catalyze the reaction of substrate hydroxylation with the subsequent oxidative decarboxylation of αKG to generate succinate and carbon dioxide [89]. Succinate, fumarate, and 2HG are both structurally and metabolically closely linked to αKG, competing with it at the active site of the enzyme. As for succinate, product inhibition additionally influences the enzymatic reaction catalyzed by αKG-dependent dioxygenases. The central roles in epigenetic control of genomic information are played by two α-KG-dependent dioxygenases, the JmjC domain-containing histone demethylases (KDMs) and the TET (ten-eleven translocation) family of DNA hydroxylases [90]. KDM act via hydroxylation of the methyl moiety within the methylated lysine residue, whereas TET enzymes demethylate 5-methylcytosine (5-mC) in a three-step oxidation reaction. First, 5mC is hydroxylated to 5-hydroxymethylcytosine (5hmC), then 5hmC is converted to 5-formylcytosine (5fC), and finally, 5fC is converted to 5-carboxylcytosine (5caC). Eventually, thymine–DNA glycosylase or other DNA repair enzymes decarboxylate 5caC, leading to DNA demethylation. Thus, inhibition of histones and DNA demethylases results in a so-called “hypermethylator phenotype”, also known as CpG island methylator phenotype (CIMP). CIMP is associated with extensive coordinated hypermethylation at specific loci and is regarded as a distinct molecular subclass of tumors in a number of human neoplasms [91].

Wild-type IDH1 and IDH2 catalyze the reversible oxidative decarboxylation of isocitrate to αKG and CO_2_. Cancer-associated mutations typically involve heterozygous mutations within the active sites of IDH1 (IDH1)^R132H^ and mitochondrial IDH2 (IDH2)^R140Q^ and (IDH2)^R172K^ [92]. *IDH1* and *IDH2* are mutated in >70% of lower-grade gliomas (grades II and III), in some glioblastomas, and ∼20% of AML, but also in cholangiocarcinoma, chondrosarcoma, and in other cases of different tumor types [92]. As a result, 2HG accumulation in cancer cells expressing mutant *IDH* results in hypermethylation of histones and CpG islands in DNA. In gliomas, it has been shown that mutation of a single gene, *IDH1*, establishes G-CIMP by remodeling and reorganization of the methylome and transcriptome [91].

Pharmacological agents that inhibit mutated IDH1 and IDH2 enzyme activity are being developed and assessed for antitumor efficacy. In the past three years, the FDA has approved two mutant IDH (mutIDH) inhibitors for relapsed or refractory AML harboring *IDH* mutation, namely enasidenib (AG-221)—which is a first-in-class oral selective inhibitor of the mutIDH2 enzyme—and ivosidenib (AG-120)-targeting mutIDH1 [93]. Positive responses to these mutIDH inhibitors were also noted in phase I/II clinical trials involving patients with relapsed or refractory gliomas, intrahepatic cholangiocarcinomas, and chondrosarcomas [94,95]. Other drugs targeting mutated enzymes producing oncometabolites are constantly being sought and tested [93].

## 9. Epigenetic Editing

For decades, the targeted manipulation of chromatin marks in living cells was unreachable. Most of the studies have used mutational approaches and pharmacological inhibition to alter epigenetic marks, but these manipulations were not target-specific and had global effects on the whole genome. Today, thanks to the so called “epigenetic editing”, it is possible to target epigenetic effector domains at any given genomic locus, making it possible to modify individual chromatin marks at a defined region and chromatin context. Most importantly, with the new methods of epigenome editing, unprecedented specificity and efficiency of the epigenetic manipulations can be achieved [96].

Epigenome-engineering tools—DNA binding proteins, such as zinc finger nucleases (ZFN) or transcription activator-like effector nucleases (TALENs) fused to epigenetic modifiers—were shown to be able to introduce the epigenetic modifications at a targeted locus. TAL effector repeats are modular DNA-binding domains that can be designed to bind essentially any genomic sequence of interest [97]. A study by Maeder et al. [98] showed that fusions of engineered transcription activator-like effector (TALE) repeat arrays and the TET1 hydroxylase catalytic domain enables efficient, targeted demethylation of specific CpGs in human cells. The authors demonstrate that these TALE-TET1 fusions enable the modification of critical methylated promoter CpGs, leading to substantial increases in gene expression. Another study showed that TALE effector can be fused to lysine-specific demethylase 1 (LSD1) to demethylate enhancer regions and to reveal enhancer target genes [99]. The fusion proteins efficiently remove enhancer-associated chromatin modifications from target loci, without affecting control regions. 

The reversible modulation of mammalian endogenous gene expression and targeted epigenetic chromatin modifications has already been demonstrated in vivo, using a mouse model. Konermann and coworkers [100] developed the Light-Inducible Transcriptional Effectors (LITEs), an optogenetic two-hybrid system integrating the customizable TALE DNA-binding domain with the light-sensitive cryptochrome 2 (CRY2) protein and its interacting partner CIB1 from Arabidopsis thaliana. Such a programmable tool was applied to a panel of 28 TALE activators as a novel mode of optogenetic control of endogenous cellular processes. Genes under LITE control showed a rise in transcription in as little as 30 min after blue light stimulation and rose steadily until saturating, with approximately 20-fold upregulation, as compared to the negative controls [100]. 

ZFN and TALENs binding specificity is determined by the amino acid sequences within their repeat domains, so changing the target region means changing the amino acid sequence [101]. Thus, the disadvantage of both ZFN- and TALENs-based epigenome-engineering tools is that the protein synthesis step is expensive, laborious, and time-consuming. Contrarily, the targeted epigenetic modification at specific genomic loci is much easier when using the clustered regularly interspaced short palindromic repeat associated protein 9 system (CRISPR/Cas9) [102]. As compared to ZFNs and TALENs, CRISPR/Cas9 is much easier to be reprogrammed to the new targets and has a significantly higher amount of targetable sites [103].

The principle of CRISPR/Cas9 used for epigenome editing purposes is based on programmable guide RNA (gRNA), catalytically dead Cas9 (dCas9), and fused (or non-covalently bound) epigenetic effector enzyme/epigenetic modifier [104]. The gRNA directs dCas9 fused to an epigenetic effector to specific loci. The effector is either the activator or repressor of gene transcription. The effectors are derived from epigenetic writers and erasers, such as DNMTs, HATs, HMTs and TETs, HDM, and HDAC, respectively [104].

CRISPR epi-editors can be divided into four distinct groups, based on their mode of action: chromatin reorganization, expression regulation, covalent histone, and DNA modification [104]. However, only the last three groups have been mostly applied so far.

Expression regulators include CRISPR activation (CRISPRa) and CRISPR interference (CRISPRi) systems. These systems consist of dCas9 and not catalytically active effector domain [104,105,106]. Their mode of action is the recruitment of either transcription promoting or repressing molecules, depending on the features of the effector. On the other hand, DNA methylation and histone modifications can be altered via epi-editors with their own enzymatic activity.

An example of a CRISPR systems that is able to modify DNA methylation at the level of a particular gene is the fusion of Tet1 or Dnmt3a with dCas9 used in a mouse model by Liu et al. [107]. They showed that targeting of the dCas9-Tet1 or -Dnmt3a fusion protein to methylated or unmethylated promoter sequences causes activation or silencing, respectively, of an endogenous reporter. In this regard, the expression of the BDNF promoter IV or the MyoD distal enhancer was restored after demethylation using dCas9-Tet1. As a consequence of the activation of MyoD expression, facilitated reprogramming of fibroblasts into myoblasts was observed. Contrarily, targeted de novo methylation of a CTCF loop anchor site by dCas9-Dnmt3a blocked CTCF binding and interfered with DNA looping. This caused altered gene expression in the neighboring loop [107]. In another study, CRISPR/dCas9 DNMT3A fusion was capable of inducing site-specific DNA methylation at the human CDKN2A and ARF promoters, and the mouse Cdkn1a promoter [108]. Importantly, the epigenome editing was efficient in regard to all three experimental procedures, and the induced methylation was sufficient to decrease the expression of all three genes.

In another study, using CRISPR/Cas9 knock-in and CRISPR/dCas9-Tet1 systems, Kang and coworkers [109] demethylated and reactivated the previously silenced Oct4 gene in NIH3T3 cells. As far as gene reactivation is concerned, the activation of *PTEN* tumor suppressor, using CRISPR/dCas9 system, was also demonstrated [110]. In this study, dCas9 was fused to the transactivator VP64-p65-Rta (VPR) in order to reactivate *PTEN* in melanoma and triple-negative breast cancer. Such a dCas9-VPR system was directed to the *PTEN* proximal promoter by gRNA, which increased *PTEN* expression, without transcriptional regulation at predicted off-target gRNA binding sites. Moreover, as a result of *PTEN* activation, downstream oncogenic pathways, including AKT, mTOR, and MAPK signaling, were significantly repressed [110].

Another example of a powerful epigenetic editing tool that can be used for gene silencing is the Krüppel associated box (KRAB) enzyme fused to dCas9. It has been shown that repression mediated by dCas9-KRAB is sufficiently specific to disrupt the activity of individual enhancers via local modification of the epigenome [111]. In this context, dCas9-KRAB was targeted to the HS2 enhancer, which is a distal regulatory element regulating the expression of globin genes. The observed effects included highly specific induction of H3K9 trimethylation (H3K9me3) at the enhancer and decreased chromatin accessibility of both the enhancer and its promoter targets. Targeted epigenetic modification of HS2 silenced the expression of multiple globin genes, and the off-target changes in global gene expression were minimal [111].

Histone modifications can also be targeted via CRISPR epi-editors. In this regard, catalytic domains of LSD1 (HDM), PRDM9 (HMT), HDAC3 (HDAC), p300 (HAT), or have been fused to ZF, TALE, or dCas9 proteins [104]. In this context, Hilton and coworkers [112] generated a programmable acetyltransferase based on the CRISPR/dCas9 fused to the catalytic core of the human acetyltransferase p300. They showed that dCas9p300 Core fusion protein acts as a potent and easily programmable tool to synthetically manipulate acetylation at targeted endogenous loci, leading to regulation of proximal and distal enhancer-regulated genes [112]. In another study, Wang et al. synthesized a group of dCas9 epi-suppressors by tethering the C terminus of dCas9 with DNMT3a, EZH2, and KRAB [113]. This epigenetic editing tool was used to target granulin (GRN), a pluripotent mitogen and growth factor promoting cancer progression by maintaining self-renewal of hepatic cancer stem cells. The study shows that the dCas9 epi-suppressors, in conjunction with gRNAs, caused significant decreases in *GRN* mRNA in Hep3B hepatoma cells. The observed effects included de novo CpG DNA methylation in the *GRN* promoter, and the production of histone codes that favor gene suppression, including decreased H3K4 methylation, increased H3K9 methylation, and enhanced HP1a binding. Eventually, the epigenetic knockdown of *GRN* led to the decreased tumor sphere formation, inhibition of cell proliferation, and reduced cell invasion [113].

Nowadays, the application of CRISPR/dCas9 in epigenetic editing of cancer cells goes beyond in vitro studies. Novel delivery systems are being created and tested in order to facilitate effective regulation of gene expression by CRISPR/dCas9 in vivo. In this regard, it has been demonstrated that a multistage delivery nanoparticle (MDNP) can achieve tumor-targeted delivery of CRISPR/dCas9 systems, restoring endogenous miRNA expression in vivo [114]. Systemic administration of MDNP/dCas9-miR-524 to tumor-bearing mice achieved effective upregulation of miR-524 in tumors. This has led to the simultaneous interference with multiple cancer related signaling pathways and remarkable tumor growth retardation [114].

Thus, epigenetic editing can be regarded as a promising approach for targeted gene therapy that is able to correct disease-associated epi-mutations. It also serves as a powerful tool to address fundamental epigenetic questions, for example, related to the cause and consequence of epigenetic marks with respect to gene expression. Nevertheless, achieving high specificity, efficient delivery, and non-immunogenicity represent the most critical challenges facing epigenome editing.

## 10. Conclusions

**Figure 1:**
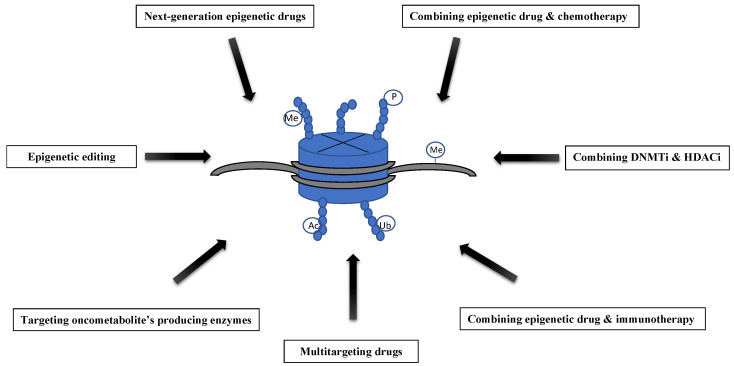
Overview of the new approaches to epigenetic therapies. Me—methylation, P—phosphorylation, Ac—acetylation, Ub—ubiquitination.

Epigenetic modifications are reversible and therefore allow excellent opportunities for therapeutic intervention. Nowadays, several epigenetic drugs are used worldwide to treat cancer. Conventional epigenetic drugs can relatively easily reach the tissues of interest, and they are proven to be effective in myelodysplastic syndromes and leukemias. Treating solid tumors with epigenetic drugs was, however, less successful. Overcoming the developing resistance and widening the therapeutic profile beyond hematological malignancies are the most important challenges faced by traditional epigenetic drugs. Finding a way for introducing more locus-specific alterations to the epigenome is another crucial issue, as traditional epigenetic drugs cause large-scale changes in gene expression, introducing not only the re-expression of genes that have been improperly silenced in cancer, but also the oncogenes and prometastatic genes. Thus novel approaches in the field of epigenetic therapy are urgently needed (Figure 1).

In order to answer these needs, next-generation epigenetic drugs are being developed and tested. Longer half-life, better bioavailability, and better safety profile are the desired features. Administration of an epigenetic drug prior to chemotherapy may be used for priming cancer cells to be more sensitive to chemotherapy, as epigenetic drugs can increase the chromatin accessibility to chemotherapeutic drugs through chromatin decompaction. Combining epigenetic drugs with chemotherapy has shown to have synergistic effects and remarkably promote potent suppression of tumorigenesis, even when tested in solid tumors. Moreover, combinations of two epigenetic drugs with different mechanisms of action (e.g., DNMTi with HDACi) represent a way to counteract chemoresistance and eventually to increase treatment efficacy. It has also been shown that epigenetic therapy plays a prominent role in modulating immune cells. However, in regard to the use of epigenetic drugs in immune-oncology, much more research has to be done in order to understand how the dosing and scheduling of these drugs in the clinical setting will modulate the immune response. Another novel therapeutic strategy for cancer treatment is the application of multitargeting drugs, i.e., drugs which target both an epigenetic enzyme, as well as other cancer-related protein. Such an approach can simplify the treatment regimens, decrease adverse drug reactions, and reduce the potential mechanisms of drug resistance.

Recently, the discovery of metabolic enzymes that can alter the epigenome has opened up a new area for drug development. Targeting metabolic causes of epigenetic changes has shown to have good clinical potential, and the first drugs with this mechanism of action have already been implemented into the clinics.

Finally, we need to emphasize that CRISPR/dCas9 technology is an extremely promising tool for targeted epigenetic therapy. It can be assumed that, with the development of CRISPR/dCas9-based technologies, the epigenetic editing field will begin to thrive.

## Figures and Tables

**Table 1:** Ongoing (recruiting or not yet recruiting) clinical trials of combined epigenetic drug (DNMTi) and other anticancer therapeutic agents (e.g., chemotherapy and immunotherapy).

Identifier	Study Title	Conditions	Epigenetic Drug	Other Anticancer Drug	Trial Phase	Estimated Enrollment
NCT04049344	Decitabine Combined with Oxaliplatin in Patients with Advanced Renal Cell Carcinoma	Metastatic Renal Cell Carcinoma	Decitabine	Oxaliplatin	II	25
NCT04510610	Camrelizumab Plus Decitabine in Anti-PD-1 Treatment-naive Patients with Relapsed/Refractory Classical Hodgkin Lymphoma	Hodgkin Lymphoma	Decitabine	Camrelizumab	II/III	100
NCT02159820	Lower Dose Decitabine (DAC)-Primed TC (Carboplatin-Paclitaxel) Regimen in Ovary Cancer	Primary Malignant Neoplasm of Ovary; FIGO Stages II to IV	Decitabine	Carboplatin-Paclitaxel	II/III	500
NCT04353479	Combined PD1 Inhibitor and Decitabine in Elderly Patients with Relapse and Refractory Acute Myeloid Leukemia	Acute Myeloid Leukemia	Decitabine	Camrelizumab (SHR-1210)	II	29
NCT03709550	Enzalutamide and Decitabine in Treating Patients with Metastatic Castration Resistant Prostate Cancer	Castration-Resistant Prostate Carcinoma, Metastatic Prostate Carcinoma in the Soft Tissue and 7 more	Decitabine	Enzalutamide	Ib/II	21
NCT02957968	Neoadjuvant Pembrolizumab + Decitabine Followed by Std Neoadj Chemo for Locally Advanced HER2- Breast Ca	Breast Adenocarcinoma; Estrogen Receptor-Negative Breast Cancer; Estrogen Receptor-Positive Breast Cancer and 10 more	Decitabine	Pembrolizumab followed by standard neoadjuvant chemotherapy	II	32
NCT03295552	Decitabine Plus Carboplatin in the Treatment of Metastatic TNBC	Metastatic Triple Negative Breast Cancer	Decitabine	Carboplatin	II	59
NCT03094637	Azacitidine and Pembrolizumab in Treating Patients with Myelodysplastic Syndrome	High Risk Myelodysplastic Syndrome, IPSS Risk Category Intermediate-1, Myelodysplastic Syndrome	Azacitidine	Pembrolizumab	II	40
NCT04490707	Study of Azacytidine Combined with Lenalidomide As Maintenance Therapy Based on MRD Monitoring in AML	Acute Myeloid Leukemia in Remission	Azacitidine	Lenalidomide	III	60
NCT03019003	Azacitidine, Durvalumab, and Tremelimumab in Recurrent and/or Metastatic Head and Neck Cancer Patients	Head and Neck Cancer	Azacitidine	Durvalumab, Tremelimumab	IB/II	59
NCT03264404	Azacitidine and Pembrolizumab in Pancreatic Cancer	Pancreas Cancer	Azacitidine	Pembrolizumab	II	31
NCT03576963	Guadecitabine and Nivolumab in Treating Refractory Metastatic Colorectal Cancer	Colorectal Adenocarcinoma, CpG Island Methylator Phenotype, Metastatic Microsatellite Stable Colorectal Carcinoma and 5 more	Guadecitabine	Nivolumab	IB/II	45
NCT03308396	Study of Durvalumab and Guadecitabine in Advanced Kidney Cancer	Advanced Kidney Cancer, Kidney Cancer, Clear Cell Renal Cell Carcinoma	Guadecitabine	Durvalumab	IB/II	58
NCT03913455	Guadecitabine in Combination with Carboplatin in Extensive Stage Small Cell Lung Cancer	Small Cell Lung Cancer, Extensive-Stage Small Cell Lung Cancer	Guadecitabine	Carboplatin	II	34

**Table 2:** Ongoing (recruiting or not yet recruiting) clinical trials of combined epigenetic drug (HDACi) and other anticancer therapeutic agents (e.g., chemotherapy and immunotherapy).

Identifier	Study Title	Conditions	Epigenetic Drug	Other Anticancer Drug	Trial Phase	Estimated Enrollment
NCT04651127	A Phase Ib/II Trial of Anti-PD-1 Antibody Combined with Histone Deacetylase Inhibitor in Patients with Advanced Cervical Cancer	Cervical Cancer	Chidamide	Toripalimab	Ib/II	40
NCT04562311	Chidamide with Immunotherapy for Patients with Locally Advanced or Metastatic Urothelial Carcinoma	Bladder Cancer Stage IV	Chidamide	Tislelizumab	II	43
NCT03820596	Sintilimab in Combination with Chidamide in Refractory and Relapsed ENKTCL	Extranodal natural killer/T cell lymphoma	Chidamide	Sintilimab	I/II	50
NCT03903458	Tinostamustine and Nivolumab in Advanced Melanoma	Malignant Melanoma	Tinostamustine	Nivolumab	I	21
NCT03024437	Atezolizumab in Combination with Entinostat and Bevacizumab in Patients with Advanced Renal Cell Carcinoma	Metastatic Cancer, Renal Cancer	Entinostat	Atezolizumab and Bevacizumab	I/II	62
NCT02616965	A Study to Assess the Feasibility of Romidepsin Combined with Brentuximab Vedotin in Cutaneous T-cell Lymphoma	Cutaneous T-cell Lymphoma (CTCL)	Romidepsin	Brentuximab vedotin	I	27
NCT03939182	Abexinostat and Ibrutinib in Diffuse Large B-cell Lymphoma and Mantle Cell Lymphoma	Diffuse Large B-cell Lymphoma and Mantle Cell Lymphoma	Abexinostat	Ibrutinib	I/II	40
NCT03829930	Combination of Entinostat and Enzalutamide in Advanced Prostate Cancer	Prostate Adenocarcinoma	Entinostat	Enzalutamide	I	18
NCT03848754	Pracinostat and Gemtuzumab Ozogamicin (PraGO) in Patients with Relapsed/Refractory Acute Myeloid Leukemia	Relapsed Adult AML	Pracinostat	Gemtuzumab Ozogamicin	I	18
NCT03742245	Olaparib in Combination With Vorinostat in Patients with Relapsed/Refractory and/or Metastatic Breast Cancer	Relapsed/Refractory and/or Metastatic Breast Cancer	Vorinostat	Olaparib	I	28

**Table 3:** The timeline of DNMTi and HDACi FDA approval. * China’s FDA approval.

	Drug Name	Year of FDA Approval
DNMTi	Azacitidine	2004
	Decitabine	2006
HDACi	Vorinostat	2006
	Romidepsin	2009
	Belinostat	2014
	Panobinostat	2015
	Chidamide *	2015

FDA = Food and Drug Administration.

## Data Availability

Not applicable.
